# Age-Related Neural Dedifferentiation in the Motor System

**DOI:** 10.1371/journal.pone.0029411

**Published:** 2011-12-22

**Authors:** Joshua Carp, Joonkoo Park, Andrew Hebrank, Denise C. Park, Thad A. Polk

**Affiliations:** 1 Department of Psychology, University of Michigan, Ann Arbor, Michigan, United States of America; 2 Center for Vital Longevity, University of Texas at Dallas, Dallas, Texas, United States of America; Katholieke Universiteit Leuven, Belgium

## Abstract

Recent neuroimaging studies using multi-voxel pattern analysis (MVPA) show that distributed patterns of brain activation elicited by different visual stimuli are less distinctive in older adults than in young adults. However, less is known about the effects of aging on the neural representation of movement. The present study used MVPA to compare the distinctiveness of motor representations in young and older adults. We also investigated the contributions of brain structure to age differences in the distinctiveness of motor representations. We found that neural distinctiveness was reduced in older adults throughout the motor control network. Although aging was also associated with decreased gray matter volume in these regions, age differences in motor distinctiveness remained significant after controlling for gray matter volume. Our results suggest that age-related neural dedifferentiation is not restricted to sensory perception and is instead a more general feature of the aging brain.

## Introduction

The dedifferentiation hypothesis of aging argues that different mental operations increasingly rely on shared neural substrates in old age [1], [2]. Consistent with this view, recent studies suggest that neural representations of visual stimuli become less distinctive with increasing age. Psychophysical studies show that aging impairs perception of moving images [3], contours [4], and object stimuli [5]. In addition, single-neuron recording studies show that visual neurons are tuned to stimulus features less selectively in older macaques than in young controls [6], [7]. Neuroimaging studies of aging humans offer the strongest evidence for this view. Brain regions that are specialized for specific categories of visual stimuli in young adults become less selective in old age [2], [8]. Furthermore, neural adaptation to face stimuli increases with age, suggesting that the aging brain is less able to differentiate one face from another [9]. Finally, distributed patterns of brain activation evoked by different visual stimuli are less distinctive in older adults than in young adults [10], [11], [12].

Although several studies have investigated age-related dedifferentiation of visual processing, less is known about the relationship between age and the neural representation of movement. Aging is associated with impaired motor performance across a range of tasks and ability domains [13], suggesting that movement representations may be disrupted in old age. Consistent with this view, older adults show stronger activation than young adults in ipsilateral motor cortex during unimanual movement [14], [15]. Older adults also show increased motor-related activation in sensory and executive regions, relative to young adults [16], [17]. Finally, motor cortical representations increase in spatial extent with age [18]. These results may reflect decreased distinctiveness of motor representations in old age. Alternatively, however, they may indicate compensation for age-related declines in cognitive or sensory function [17], [19].

Thus, the present study investigated the effects of aging on the neural representation of movement. Previous studies of the aging motor control system have focused on univariate measures, which may not capture fine-grained spatial information patterns that discriminate between task conditions. Thus, we assessed the distinctiveness of motor representations in young and older adults using multi-voxel pattern analysis (MVPA), which is more sensitive to such patterns [20]. According to the dedifferentiation hypothesis, the neural representations of different motor states should be less distinctive in older adults than in young adults [1]. We define the representation of a particular motor state as the distributed pattern of neural activation evoked by that state [21]; the representations of two motor states are distinctive to the extent that one pattern can be distinguished from the other. Thus, we predicted that the multi-voxel activation patterns evoked by left- and right-hand finger tapping would be less distinctive in older adults, relative to young adults.

## Methods

### Ethics statement

All study procedures were reviewed and approved by the University of Illinois Institutional Review Board, and all participants provided detailed written consent before their involvement in this study according to the principles of the Declaration of Helsinki.

### Participants

Twenty-four older adults and twenty-three young adults participated in the experiment. Data from five older adults and four young adults were discarded due to excessive head motion, improper head coil placement, vision problems, or failure to follow instructions, leaving data from eighteen older adults (mean age: 64.67; standard deviation: 2.9; range: 60–69; nine female) and nineteen young adults (mean age: 22.2; standard deviation: 2.7; range: 18–29; 9 female) for analysis. All participants were right-handed native English speakers; participants were not taking medications with psychotropic or vascular effects, and were free of MRI safety contraindications. All participants scored at least 26 on the mini-mental state exam [22].

### Experimental design

Participants performed simple motor and visual tasks while fMRI data were collected. The motor task comprised two six-minute runs. In each block, subjects were instructed to tap their left index finger (three blocks per run), right index finger (three blocks per run), or to alternate between left and right index fingers (six blocks per run). Large red arrows were used to cue each condition. Participants tapped in time with a loud 1 Hz metronomic tick presented through the scanner intercom. Blocks were presented in one of two possible fixed orders, either (1) left finger, alternate, right finger, alternate, etc., or (2) right finger, alternate, left finger, alternate, etc.; block orders were counterbalanced across runs and subjects. Each block lasted for 30 seconds; there was no gap between blocks. An independent analysis of the visual task, which does not overlap with the present study, has been published in a separate report [11].

Stimuli were presented using E-prime (Psychology Software Tools, Pittsburgh, PA) and displayed using a back-projection system. Responses were recorded using a Lumina response pad (Cedrus Corporation, San Pedro, CA).

### Data acquisition

Brain images were acquired using a 3T Allegra head-only MRI scanner (Siemens, Erlangen, Germany). Blood oxygen level dependent (BOLD) images were acquired using an echo planar imaging sequence (TR = 2000 ms, TE = 25 ms, FA = 80°, FOV = 220 mm). Each volume included 36 axial slices collected parallel to the AC–PC line. Each slice was 4.4 mm thick, with an in-plane resolution of 3.44 by 3.44 mm. A high resolution (1 mm isotropic voxels) T1-weighted MPRAGE image was also collected for subsequent normalization to standard space.

### Pre-processing

Data were pre-processed using SPM8 software (Wellcome Department of Cognitive Neurology, London, UK) running under Matlab R2011b (The Mathworks, Inc., Natick, MA, USA). Functional images were corrected for slice timing, realigned to the first functional volume, and coregistered to the high-resolution structural image. Spatial normalization and smoothing may distort or remove fine-grained information from multivariate analysis [20]. Thus, neither normalization nor smoothing was applied before multivariate analysis.

### Model estimation

Neural responses were estimated using the General Linear Model, implemented in SPM8. Responses to the left- and right-hand tapping conditions were modeled using a block design; the alternation condition was not explicitly modeled but was treated as an implicit baseline. Model estimation included twenty-four head motion regressors as nuisance covariates, including the linear, squared, time-shifted, and squared time-shifted transformations of the six rigid-body movement parameters.

### Multi-voxel pattern analysis

Next, we used the activation estimates from the univariate analysis described above to assess the distinctiveness of multi-voxel representations of left- and right-hand tapping. As described by Haxby and colleagues [23], neural distinctiveness was defined as the difference between pattern similarity within and between conditions. Specifically, the distinctiveness between conditions for a given set of voxels was defined as the difference between the mean Fisher-transformed Pearson correlations across those voxels' activation values within and between the two conditions [23], [24]. Positive distinctiveness scores (i.e., greater within-condition than between-condition similarity) indicate that multi-voxel activation patterns distinguished between conditions; distinctiveness scores of zero indicate that activation patterns were similar across conditions. We chose this approach over alternative classification methods, such as support vector machines and artificial neural networks, because of its computational simplicity and to avoid ceiling effects in classifier accuracy.

To generate whole-brain maps of pattern distinctiveness, we combined the correlation analysis described above with a multivariate searchlight procedure [25]. For each voxel in the brain, we identified all voxels within a 12-mm-radius sphere centered on that voxel. Next, we estimated the distinctiveness between conditions across this group of voxels. The resulting distinctiveness score was then entered as the value for the center voxel. This procedure was repeated for each voxel in the brain, yielding a whole-brain map of distinctiveness between conditions. Neural distinctiveness maps were subsequently normalized into Montreal Neurological Institute (MNI) space for further analysis.

### Voxel-based morphometry

Gray matter volume declines with increasing age in regions associated with motor control, including the cerebellum and caudate [26]. Recent research shows that these age-related changes in brain structure may explain age differences in brain function [27]. Thus, the present study also investigated whether age differences in the distinctiveness of motor representations could be explained by differences in gray matter volume. Voxel-based morphometry (VBM) was implemented using the VBM8 toolbox for SPM8 (http://dbm.neuro.uni-jena.de/vbm.html). High-resolution anatomical images were segmented, modulated using the non-linear warping parameters from the normalization results, and smoothed with a Gaussian kernel of 8 mm full width at half maximum.

## Results

**Figure 1:**
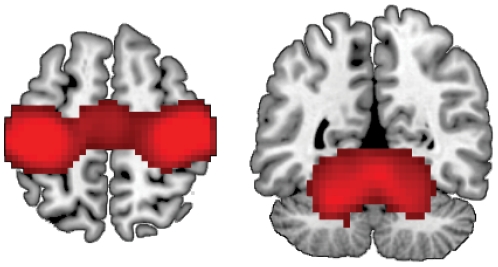
Whole-brain searchlight analysis of the distinctiveness of motor representations, collapsing across age. Distributed patterns of activation in primary motor cortex, pre-supplementary motor area (left panel; z = 56) cerebellum (right panel; y =  −52) reliably distinguished between left- and right-hand finger tapping. Coordinates are reported in MNI space.

First, we identified the brain regions in which multi-voxel patterns distinguished between left- and right-hand finger tapping conditions using a whole-brain searchlight procedure, collapsing across age groups. This analysis used a height threshold of *p*≤1e−7 and an extent threshold of k≥50 voxels. Results indicated that distributed patterns of activation in bilateral primary motor cortex (M1), supplementary motor cortex (SMA), and medial and lateral cerebellum distinguished between conditions (Table 1, Figure 1).

**Table 1:** Whole-brain searchlight analysis of motor representational distinctiveness, collapsing across age.

Brain regions	Number of voxels	MNI coordinates			Peak t-score
		X	Y	Z	
L. motor cortex	2202	−43	−26	56	18.33
R. motor cortex	2202	43	−19	56	18.80
Pre-supplementary motor area	2202	2	−13	60	10.13
L. cerebellum	1207	−22	−50	−28	15.04
M. cerebellum	1207	2	−57	−15	14.23
R. cerebellum	1207	22	−50	−28	11.33

**Figure 2:**
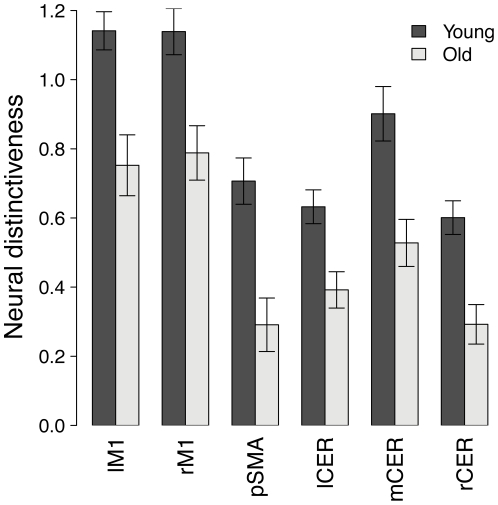
Region-of-interest analysis of neural distinctiveness in the motor network. Neural distinctiveness was reduced throughout the motor network in older adults, relative to young adults. Error bars denote the standard error of the mean.

**Figure 3:**
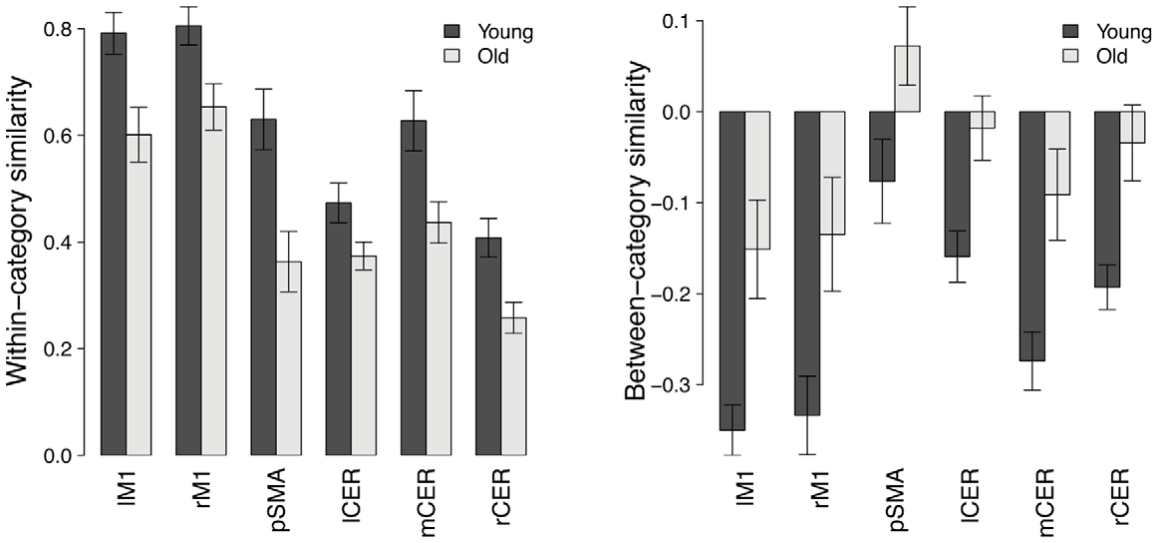
Region-of-interest analysis of within- and between-category similarity in the motor network. Older adults showed reduced within-category similarity (left panel) and increased between-category similarity (right panel) throughout the motor network. Error bars denote the standard error of the mean.

Next, we compared neural distinctiveness across age groups in each region highlighted by the preceding searchlight analysis. Regions of interest were defined as spheres of 6 mm in radius centered on the local maxima of the searchlight map. In each region, the distinctiveness of activation patterns evoked by left- and right-hand tapping was significantly lower in older adults than in young adults (Figure 2; left M1: *t*(35) = 3.79, *p*<0.001; right M1: *t*(35) = 3.41; *p* = 0.0016; SMA: *t*(35) = 4.08, *p*<0.001; left cerebellum: *t*(35) = 3.36; *p* = 0.0019; right cerebellum: *t*(35) = 4.13, *p*<0.001; medial cerebellum: *t*(35) = 3.57, *p* = 0.0011). Age differences in neural distinctiveness were driven by changes in both within- and between-condition similarity: older adults showed decreased within-category similarity (Figure 3, left panel; left M1: *t*(35) = 2.97, *p* = 0.0053; right M1: *t*(35) = 2.71, *p* = 0.010; SMA: *t*(35) = 3.32, *p* = 0.0021; left cerebellum: *t*(35) = 2.15, *p* = 0.038; right cerebellum: *t*(35) = 3.20, *p* = 0.0029; medial cerebellum: *t*(35) = 2.75, *p* = 0.0093) and increased between-category similarity (Figure 3, right panel; left M1: *t*(35) = 3.32, *p* = 0.0021; right M1: *t*(35) = 2.64, *p* = 0.012; SMA: *t*(35) = 2.35, *p* = 0.025; left cerebellum: *t*(35) = 3.14, *p* = 0.0034; right cerebellum: *t*(35) = 3.32, *p* = 0.0021; medial cerebellum: *t*(35) = 3.11, *p* = 0.0037) in all regions of interest.

Next, we assessed the contributions of structural changes to the age differences in neural distinctiveness described above using voxel-based morphometry (VBM). In each region of interest, gray matter volume was significantly reduced in older adults, relative to young adults (left M1: *t*(35) = 7.81, *p*<0.001; right M1: *t*(35) = 7.60, *p*<0.001; SMA: *t*(35) = 6.20, *p*<0.001; left cerebellum: *t*(35) = 4.74, *p*<0.001; right cerebellum: *t*(35) = 3.61, *p*<0.001; medial cerebellum: *t*(35) = 4.15, *p*<0.001). However, after controlling for individual differences in gray matter volume, age differences in neural distinctiveness remained highly significant in left primary motor cortex (*t*(35) = 2.49, *p* = 0.018), supplementary motor area (*t*(35) = 3.22, *p* = 0.0028), lateral cerebellum (left: *t*(35) = 3.56, *p* = 0.0011); right: *t*(35) = 3.80, *p*<0.001), and medial cerebellum (*t*(35) = 2.81, *p* = 0.0081); the age difference in right primary motor cortex was no longer significant (*t*(35) = 1.16, *n.s.*).

**Figure 4:**
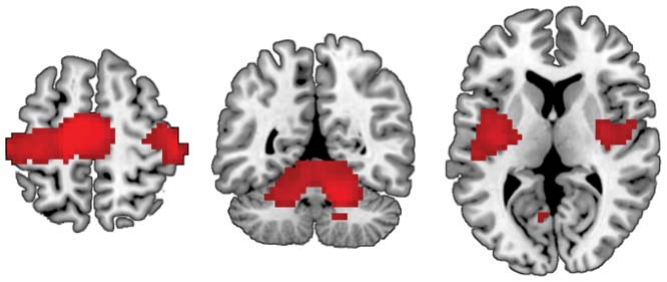
Whole-brain searchlight analysis of age differences in motor distinctiveness. Neural distinctiveness was significantly higher in young adults than in older adults in primary motor cortex, pre-supplementary motor area (left panel; z = 56), cerebellum (center panel; y =  −52), and insula (right panel; z = 8). Coordinates are reported in MNI space.

Finally, we conducted an exploratory whole-brain analysis of the effects of age group on neural distinctiveness. This analysis used a height threshold of *p*≤0.005 and an extent threshold of k≥50 voxels. Results confirmed that distinctiveness was reduced in older adults throughout the motor execution network. Furthermore, we also observed decreased neural distinctiveness among older adults in bilateral insula (Table 2, Figure 4). No regions showed greater distinctiveness for older adults than for young adults.

**Table 2:** Whole-brain searchlight analysis of age differences in motor distinctiveness.

Brain regions	Number of voxels	MNI coordinates			Peak t-score
		X	Y	Z	
L. motor cortex	967	−36	−16	60	3.85
R. motor cortex	242	50	−13	60	4.43
Pre-supplementary motor area	967	−15	−16	56	6.08
L. cerebellum	752	−22	−50	−28	3.52
M. cerebellum	752	−2	−61	−6	4.21
R. cerebellum	752	19	−44	−28	4.88
L. insula	323	−36	−9	3	4.17
R. insula	176	36	−9	12	3.56

## Discussion

The dedifferentiation hypothesis of cognitive aging argues that representations of different mental states become more similar with increasing age [1]. Recent neuroimaging studies of visual perception support this view, indicating that distributed patterns of brain activation evoked by different visual stimuli are less distinctive among older adults than young adults [10], [11]. A range of motor skills, including movement speed, coordination, and postural stability, decline with increasing age [13]. Such findings suggest that the distinctiveness of motor representations may also decrease in old age. However, studies of the effects of aging on representational distinctiveness have focused on perception; less is known about the relationship between age and motor representations.

The present study used multi-voxel pattern analysis (MVPA) to investigate the effects of age on the distinctiveness of motor representations. We found that motor distinctiveness was reduced among older adults in primary motor cortex, the supplementary motor area, the insula, and the cerebellum. No brain regions showed greater distinctiveness for older adults than young adults, suggesting that older adults do not compensate for decreased motor distinctiveness by extending motor representations to additional brain regions. Thus, previous reports of age-related over-activation during motor performance [15], [17] may reflect compensation for motor deficits via the recruitment of additional cognitive control resources that do not directly encode motor actions. In other words, although previous studies indicate that older adults can indeed compensate for declining neural function, our results imply that this compensation does not involve the extension of distinctive motor representations to additional regions not recruited by young adults. Finally, although we observed age-related losses of gray matter volume in regions related to motor control, these differences in brain structure did not account for age-related declines in motor distinctiveness.

Our results provide novel support for the dedifferentiation hypothesis. In particular, we found that age-related neural dedifferentiation characterizes the representation of action as well as perception. Recent studies of animals suggest that neural specialization may decline with age in the auditory [28] and somatosensory domains as well [29]; future studies might conduct complementary tests in aging humans. In addition, little is known about the causes of age-related dedifferentiation. Park and colleagues [30] argue that dedifferentiation in the visual system reflects broadened tuning curves in some brain regions and attenuated activation in others; future research should investigate the contributions of age-related broadening and attenuation to dedifferentiation of the motor cortex.

Recent studies have also linked dedifferentiation to age differences in neurotransmitter function. For example, Li and colleagues [1] have hypothesized that dedifferentiation reflects age-related declines in dopamine availability, arguing that decreased dopamine function leads to increased neural noise in old age. Indeed, older adults with greater dopamine transporter binding exhibit faster simple reaction times [31], and treatment with the dopamine precursor levodopa improves motor performance in the elderly [32]. Age-related declines in motor representations may also be accelerated in movement disorders like Parkinson's disease [13]. In addition, recent studies have linked age differences in GABA-ergic inhibition to declining neural selectivity. In particular, age-related visual impairments are accompanied by selective losses of GABA-reactive neurons in cats [33], and increased GABA availability is associated with improved motor control in humans [34]. Age differences in dopamine, GABA, and other neurotransmitter systems may also exert interactive effects on motor representation and motor performance. Future research should continue to explore the neurochemical origins of age-related dedifferentiation.

The present findings also highlight the complexity of structure-function relationships across the lifespan. Although age-related declines in brain structure integrity explain age differences in activation in certain brain regions during certain tasks [27], the present results show that age differences in the distinctiveness of motor and visual representations are not explained by differences in brain structure. Future research might investigate the contexts in which developmental differences in brain function can, and cannot, be attributed to differences in brain structure.

Although the present study was designed to test theoretical models of cognitive aging, our findings also have important implications for applied research. In particular, our results suggest that brain-computer interface (BCI) devices may be less effective in older adults than in young adults. These devices often rely on neural signals related to motor execution or imagery, and, as such, require that different motor states correspond to distinctive neural representations. The present finding of reduced motor distinctiveness in older adults thus implies that the performance of BCI systems tested on healthy young adults will likely degrade when used with older patients.

Interpretation of the present results is constrained by a number of limitations that we hope will be addressed in future studies. For example, our sample included young and older adults, but not middle-aged adults. Thus, we cannot yet determine whether age-related changes in motor representations progress gradually over time or onset rapidly in old age. Furthermore, because the present study used a simple unimanual finger tapping task, we were unable to assess the effects of aging on the representation of complex movements. Finally, because we used a block design, we were unable to examine the time-course of neural responses to individual movements. Thus, future studies using middle-aged subjects, more complex movement tasks, and event-related task designs could considerably expand our understanding of age differences in movement representations.

In sum, our findings provide new support for the dedifferentiation hypothesis of aging, showing that neural representations of motor actions grow less distinctive in old age. Further, our findings raise new questions about the generality and causes of age differences in neural representation. Finally, the present study highlights the value of multivariate analytic techniques for the study of group differences in neural representation.
